# Changes in Selected Food Groups Consumption and Quality of Meals in Japanese School Children during the COVID-19 Pandemic

**DOI:** 10.3390/nu13082743

**Published:** 2021-08-10

**Authors:** Chika Horikawa, Nobuko Murayama, Yui Kojima, Hisako Tanaka, Naho Morisaki

**Affiliations:** 1 Department of Health and Nutrition, University of Niigata Prefecture Faculty of Human Life Studies, 471 Ebigase, Higashi-ku, Niigata 950-8680, Japan; murayama@unii.ac.jp (N.M.); y_kojima@unii.ac.jp (Y.K.); 2 Department of Social Medicine, National Center for Child Health and Development, 2-10-1 Okura, Setagaya-ku, Tokyo 157-8535, Japan; tanaka-hs@ncchd.go.jp (H.T.); morisaki-n@ncchd.go.jp (N.M.)

**Keywords:** COVID-19, household income, quality of meals, animal protein sources, fruit, vegetables, burden for preparing meals, schoolchildren, Japan

## Abstract

In 2020, a state of emergency was declared to control the devastating impact of coronavirus, leading to temporary school closures in Japan, meaning that school lunches were not provided to the majority of schoolchildren. Using questionnaires completed by participants’ guardians, we examined the relationship between household income and the quality of meals in Japanese schoolchildren before, during, and after the state of emergency. Participants (1111 children, 10–14 years old) were chosen to form a nationally representative sample of the Japanese population. “Well-balanced dietary intake” was defined as the intake of (i) meat, fish, or eggs and (ii) vegetables. The desired prevalence was defined as equal to or more than twice a day. Household income was divided into quartiles. “Well-balanced dietary intake” was lower in all households during the state of emergency compared with before. The proportion of those with a “well-balanced dietary intake” at least twice a day was notably low in both Q3 and Q4 during the state of emergency compared with before the declared state of emergency; relative risk increase (95% CI) were Q1: −19.0% (−19.6% to −18.4%), *p* < 0.001, Q2: −21.3% (−22.1% to −20.6%) *p* < 0.001, Q3: −25.4% (−26.1% to −24.7%), *p* < 0.001, and Q4: −34.8% (−35.6% to −34.0%), *p* < 0.001. The interaction *p* (vs. Q1) of Q2, Q3, and Q4 were all <0.001. Guardians from low-income households had significantly higher rates of having less: time, psychological room, and financial position to prepare meals during the state of emergency. Our results suggest that schoolchildren’s quality of meals worsened during the state of emergency, especially in low-income households, because school lunches were not provided.

## 1. Introduction

The coronavirus disease 2019 (COVID-19) pandemic, caused by severe acute respiratory syndrome coronavirus 2 (SARS-CoV-2) [1], continues to spread worldwide, posing a serious threat to our healthcare and causing social and economic impacts everywhere [2,3,4]. The Japanese Government declared a nationwide state of emergency on 16 April 2020, based on the Act on Special Measures for Pandemic Influenza and New Infectious Diseases Preparedness and Response (Act No. 31 of 2012) [5]. From 16 April to 6 May 2020, each prefecture was asked to cooperate in restricting the use of educational facilities: 95% of elementary and junior high schools in Japan implemented a temporary closure on 22 April, with the aim of controlling the spread of the infection as quickly as possible [6,7]. One of the impacts on children was the loss of school lunches during the COVID-19 state of emergency.

During the fiscal year 2018, the Japanese Government provided school lunches to 99.1% of schoolchildren in their elementary years (6–11 years of age) and 89.9% in their junior high years (12–15 years of age). School lunches are provided on weekdays, which equates to approximately 190 days per year [8]. School lunches were not provided in schools that were temporarily closed during the COVID-19 state of emergency: schoolchildren were required to have their lunch at home during this period. The School Lunch Program Act was enacted in 1954 to improve children’s nutrition [9,10,11]. The Standards for the School Lunch Program requires school lunches to contain at least a third of children’s daily energy and nutritional requirements, including vitamin A (≥40%), vitamin B1 (≥40%), vitamin B2 (≥40%), Ca (≥50%), Fe (≥40%), and fiber (≥40%) [11]. Therefore, it is important to determine the quality of meals for schoolchildren when their opportunities for lunch depend on the home.

It is well known that socioeconomic status (SES) is related to nutritional intake status in schoolchildren, which, in turn, affects children’s growth, including biological and cognitive changes [12,13]. A systematic review of Western children revealed that low SES is positively associated with a low intake of several vitamins and minerals [14]. In addition, a Korean study showed that children in low SES households had reduced consumption of energy from protein and increased energy from carbohydrates when compared with children in high SES households [15]. We previously reported that Japanese schoolchildren from low-income households had high carbohydrate intake levels and low intake levels of fish/shellfish, sugar, protein, and several micronutrients [16]. The school lunch is an important meal and system for correcting disparities in food groups and nutrient intake among children which may occur due to the household income gap [16,17]. We also reported that, on days when school lunches were not provided, vitamin B6, pantothenic acid, K, Mg, P, Fe, and Zn levels were significantly lower in Japanese children from low-income households when compared with children from middle-income households; the differences were not significant on days when school lunches were provided [17]. The results suggest it is necessary to examine whether the low quality of meals is also observed in children from low-income households during the COVID-19 state of emergency, when school lunches were not provided.

In this study, we assessed the quality of meals eaten by schoolchildren and investigated whether the quality differs, depending on household income before, during, and after the COVID-19 state of emergency. We also examined whether there is a relationship between household income and the guardians’ burden for preparing meals during the pandemic.

## 2. Materials and Methods

### 2.1. Study Participants

A stratified two-stage clustering design was used to obtain a nationally representative sample of households in Japan; the sampling frame used the resident registration system from the fiscal year 2020. The Residential Basic Book Act stipulates that all residents of Japan must be registered. We used the 8 regions of Japan (Hokkaido and Tohoku, Kanto, Hokuriku koushinetsu, Chubu, Kinki, Chugoku, Shikoku and Kyushu, and Okinawa) for stratification. A random sample of 6–7 municipalities was drawn from each prefecture, producing a total of 50 municipalities in the first sampling stage. In the second sampling stage, 30 households with children in the fifth grade of elementary school (10–11 years old) or the second grade of junior high school (13–14 years old) were randomly selected from each municipality; a total of 3000 households were extracted. Questionnaires were sent to the guardians of schoolchildren in December 2020, and the responses were collected by mail. The deadline for responses was presented as 3 weeks. Reminder letters were sent to each household 1 week after the questionnaires were sent. Households who answered the questionnaires were sent a Quo card worth 600 JPY as a reward. This study was conducted in accordance with the Declaration of Helsinki and the Ethical Guidelines for Clinical/Epidemiological Studies of the Japanese Ministry of Health Labor and Welfare. Ethical approval was obtained from the ethics committee of the National Center for Child Health, Japan (No. 2020-168) and the University of Niigata Prefecture, Japan (No. 2025). Written informed consent was obtained for all subjects. Out of 3000 households invited to join the study, 1551 (51.7%) households responded to the invite, but only 1111 households (37.0%) completed the questionnaire. All completed questionnaires were included in the final analysis.

### 2.2. Household Socioeconomic Status

Household income levels were determined for all household members and included salary, benefits, family allowance, and rental income during 2020. Participants selected one of the following answers: (i) <1 million JPY, (ii) between 1 and 2 million JPY, (iii) between 2 and 3 million JPY, (iv) between 3 and 4 million JPY, (v) between 4 and 5 million JPY, (vi) between 5 and 6 million JPY, (vii) between 6 and 7 million JPY, (viii) between 7 and 8 million JPY, (ix) between 8 and 10 million JPY, (x) ≥10 million JPY, or (xi) do not wish to answer. Equivalent household income was calculated by dividing each household income by the square root of the number of household members [18]. Representative incomes (0.5, 1.5, 2.5, 3.5, 4.5, 5.5, 6.5, 7.5, 9, and 11 million JPY) were determined from the midpoint of each income category [19]. We divided the income variables into quartiles of household income levels: Q1 (highest income level, *n* = 342), Q2 (*n* = 251), Q3 (*n* = 288), and Q4 (lowest income level, *n* = 230). Equivalent income adjusted for the number of household members was divided into quartiles for each municipality from which the participants were selected. The number of participants assigned to Q1–4 was not necessarily the same because there were several subjects with the same income level. The mean incomes of each group were: 5.0 ± 1.0, 3.6 ± 0.8, 2.8 ± 0.9, and 1.7 ± 0.9 million JPY, respectively. The poverty line in Japan for the year 2018 was 1.3 million JPY, and the ratio of people living below the poverty line was 15.4%, according to a report from the Ministry of Health, Labour and Welfare of Japan [20]. Therefore, Q4 falls into the household income level near the poverty line. The questionnaire also determined the total number of people living in the household and the educational attainment of each guardian.

### 2.3. Intakes of Selected Food Groups of School Children and Burden for Preparing Meals

Intakes of selected food groups of the schoolchildren were determined before, during, and after COVID-19 state of emergency conditions and were assessed by the prevalence of consuming the following foods at least twice a day: 1. milk and dairy products, 2. meat, fish, or eggs, 3. vegetables, and 4. fruits. The prevalence of these foods eaten during the periods before, during, and after the declaration of the state of emergency was determined. The details of these questions are shown in Table 1. A Japanese-style diet consists of meals that include grains, protein-rich foods such as fish and meat, and vegetables [21,22]. In Japan, the basic staple food is rice [23,24], while fruit intake is relatively low when compared with other developed countries: about 1/3 to 1/2 lower than the intake in Western countries [24]. In this study, a “well-balanced dietary intake” was defined as the intake of (i) meat, fish, or eggs and (ii) vegetables. We also assessed the prevalence of eating these foods; the desired prevalence was defined as equal to or more than twice a day. In a previous study, people who frequently consumed (≥1.75 times/day) a well-balanced diet had high levels of protein, polyunsaturated fat, n-6 and n-3 polyunsaturated fat, total dietary fiber, soluble and insoluble dietary fiber, β-carotene, α-tocopherol, vitamin K, thiamin, riboflavin, folate, pantothenic acid, vitamin C, potassium, calcium, magnesium, iron, and copper, and lower levels of carbohydrate [21]. They also had a lower risk of not meeting the recommended dietary allowance for vitamin A, vitamin C, and calcium, and had an adequate intake of potassium when compared with participants in the low-frequency group for “well-balanced dietary intake” [22]. According to a report from the Ministry of Environment in Japan, the mean percentage of food waste from Japanese schoolchildren’s school lunches was 6.9% [25]. School lunches count as one serving of “well-balanced dietary intake” [8], and all schoolchildren are considered to be provided with 2. (meat, fish, or eggs) and 3. (vegetables) by having school lunches [8]. 

The burden for preparing meals was assessed before and after the COVID-19 state of emergency was declared using the following statements on the questionnaire: (i) I have less time to prepare meals than before the state of emergency, (ii) I have more time to prepare meals than before the state of emergency, (iii) I have less psychological room to prepare meals, (iv) I have more psychological room to prepare meals, (v) I have a lesser financial position for choosing or consuming foods and meals, and (iv) the above situations are not applicable to me.

### 2.4. Sociodemographics and Anthropometrics Data

The questionnaire also asked for information on gender, economical circumstance of life after COVID-19 state of emergency, and educational attainment levels of the guardians. Participants were asked to select from the following statements to best explain their circumstance of life after the COVID-19 state of emergency: (i) worse than before the state of emergency, (ii) no different than before the state of emergency, (iii) better than before the state of emergency, and (iv) do not want to answer. The education attainment levels were selected from (i) less than high school, (ii) high school, (iii) vocational, (iv) junior college, (v) university/graduate school, or (vi) child does not have a father or mother (vii) do not know/do not want to answer. The height and body weight of each child was also obtained by self-reporting from each guardian as of December 2020. Body mass index (BMI) was then calculated. Height, weight, and BMI percentiles are the recommended values from The Japanese Society of Pediatric Endocrinology and The Japanese Association for Human Auxology [26,27,28].

### 2.5. Statistical Analysis

The weights of participants were grouped into their municipality so that the different areas of residence were used to represent the general population. Categorical variables were expressed as a percentage of participants; numerical variables were expressed as means and standard deviation (SD) or 95% confidence interval (CI). The χ^2^ test was used to compare categorical data among household incomes, and numerical variables were examined using one-way analysis of variance. We assessed whether there was any difference in preparing meals according to household income before and after the COVID-19 state of emergency. Poisson regression analyses were used to estimate relative risk increases (RRIs) and 95% CIs by conducting a quartile analysis: we assigned the highest income level (Q1) as the referent. To assess whether schoolchildren’s prevalence of having a “well-balanced dietary intake” at least twice a day differed, according to household income during and after COVID-19 state of emergency, Poisson regression analyses were used to estimate the RRIs and 95% CIs by dividing household SES into quartiles, and “before” COVID-19 state of emergency was used as the reference for “during” or “after” COVID-19 state of emergency. Adjustments were made for gender, age (10–11- or 13–14 years old), and BMI (continuous). Further analyses also adjusted for the education attainment levels of the male and female guardians. All *p*-values were two-sided, and the significance level was <0.05. All statistical analyses were performed using SPSS Statistics Version 27 (IBM, Armonk, NY, USA).

## 3. Results

The characteristics and household incomes of the group containing 10–14-year-old schoolchildren (1111 participants; 49.1% boys, mean BMI; 18.6 ± 3.1 kg/cm^2^) are shown in Table 2. Significant differences were observed among the four income groups in terms of gender, number of family members, annual household income, the economical circumstance of life after COVID-19 state of emergency, and educational attainment levels of the guardians (all *p* < 0.001). The prevalence of participants who answered “Economical circumstance of life after COVID-19 state of emergency is worse than before the state of emergency” was 13.6% for those in Q1 (highest income level), 14.5% for Q2, 31.6% for Q3, and 49.0% for Q4 (lowest income level. Height, weight, and BMI distributions of study participants were similar to representative distributions of Japanese children [26,27,28]; however, distribution differed by household income level (*p* < 0.001). The prevalence of children with high (>97%) or low (3%) values was higher among children in lower-income households.

Table 3 shows the quality of meals for schoolchildren according to household income level before, during, and after the COVID-19 state of emergency. The prevalence of intake of milk and dairy products, meat, fish, or eggs, vegetables, and fruits was lower in schoolchildren in all four household income levels during the state of emergency compared with before. Prevalence of intake was also higher after the COVID-19 state of emergency compared to during the state of emergency. The prevalence of intake was similar before and after the state of emergency in all four household income levels. 

**Figure 1:**
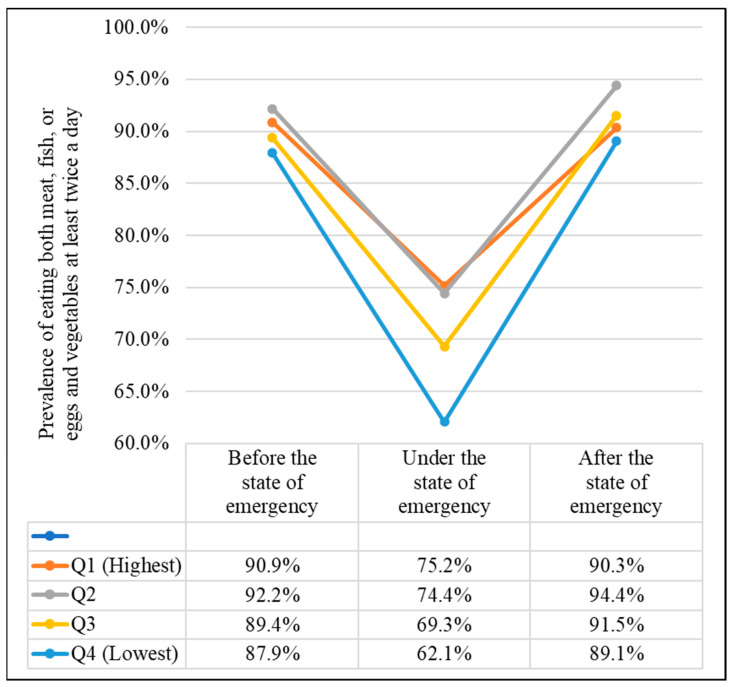
Household income level and prevalence of “well-balanced dietary intake” at least twice a day: 10–14-year-old schoolchildren in Japan before, during, and after COVID-19 state of emergency “Well-balanced dietary intake” was defined as the intake of both meat, fish, or eggs and vegetables.

Table 4 and Figure 1 show the results of the associations between household income level and prevalence of “well-balanced dietary intake” in Japanese schoolchildren before, during, and after the COVID-19 state of emergency. The percentage of respondents who consumed a well-balanced diet, at least twice a day, was significantly lower during the state of emergency compared with those before the state of emergency in all income quartiles (RRI (95% CI). Q1: −19.0% (−19.6% to −18.4%), *p* < 0.001, Q2: −21.3% (−22.1% to −20.6%) *p* < 0.001, Q3: −25.4% (−26.1% to −24.7%), *p* < 0.001, and Q4: −34.8% (−35.6% to −34.0%), *p* < 0.001, using confounder-adjusted Poisson regression analyses). Additionally, the lower the income group, the greater the rate of decrease from before the state of emergency: *p* (vs. Q1) of Q2, Q3, and Q4 were all < 0.001. After the state of emergency, there were significant differences in the percentage of people who consumed a well-balanced diet, at least twice a day, compared with before the state of emergency, in all of the income quartiles; however, the differences were small (Q1: −0.6% (−1.2% to 0.0%), *p* = 0.042, Q2: 2.4% (1.7% to 3.1%), *p* < 0.001, Q3: 2.4% (1.8% to 3.0%), *p* < 0.001, and Q4: 1.3% (0.6% to 2.0%), *p* < 0.001). The proportion in Q4 with a “well-balanced dietary intake”, at least twice a day, was particularly low compared to Q1, Q2, and Q3, before, during, and after the state of emergency. Furthermore, the proportion of those consuming “well-balanced dietary intake” at least twice a day was notably low in both Q3 and Q4 during the declared state of emergency (Q1: 75.2% Q2: 74.4%, Q3: 69.3%, and Q4: 62.1%).

Table 5 shows the association between household income level and the guardians’ subjective sense of burden for preparing meals after the COVID-19 state of emergency. Participants in Q3 and Q4 had significantly higher rates of answering “I have less time to prepare meals than before the state of emergency” (Q3: 31.4% (29.9% to 33.0%), *p* < 0.001, and Q4: 28.2% (26.5% to 29.9%), *p* < 0.001); however, participants in Q2 had a significantly lower rate compared with Q1 (−10.9% (−12.7% to −9.1%), *p* < 0.001, using Poisson regression analysis). Participants from households with lower incomes (Q2-4) had significantly lower rates of answering: “I have more time to prepare meals than before the state of emergency” (Q2: −30.6% (−32.0% to −29.3%), *p* < 0.001, Q3: −44.0% (−45.4% to −42.7%), *p* < 0.001, and −45.7% (−47.1% to −44.2%), *p* < 0.001), and “I have more psychological room to prepare meals” (Q2: −71.3% (−73.1% to −69.4%), *p* < 0.001, Q3: −59.0% (−60.7% to −57.3%), *p* < 0.001, and Q4: −36.9% (−38.6% to −35.3%), *p* < 0.001). In addition, they had significantly higher rates of answering “I have less psychological room to prepare meals” (Q2: −71.3% (−73.1% to −69.4%), *p* < 0.001, Q3: −59.0% (−60.7% to −57.3%), *p* < 0.001, and Q4: −36.9% (−38.6% to −35.3%), *p* < 0.001) and “I have less time to prepare meals than before the state of emergency” (Q2: (91.0% to 96.4%) *p* < 0.001, Q3: 167.5% (165.1% to 169.9%), *p* < 0.001, and Q4: 229.9% (227.5% to 232.2%)) compared with those from higher income households (Q1). 

## 4. Discussion

The COVID-19 global pandemic continues to have a serious impact on the lives of both adults and children [2,3,4]. During the COVID-19 state of emergency in Japan, school lunches were not provided to most Japanese schoolchildren due to nationwide temporary school closures [7]. This is the first study to measure the association between the quality of meals for schoolchildren and household income before, during, and after the COVID-19 state of emergency. During the state of emergency, the prevalence of “well-balanced dietary intake” in Japanese schoolchildren was lower compared to before and returned to the same prevalence after the state of emergency had ended, regardless of household income levels. The prevalence of intake of milk and dairy products, meat, fish, or eggs, vegetables, and fruits during the state of emergency was lower compared to before the state of emergency in all four household income levels; intake returned to the same prevalence as before, when the state of emergency was declared over. The decrease in prevalence during the state of emergency was larger among low-income households. Our results show that the quality of meals for schoolchildren was reduced during the state of emergency, suggesting that school lunches are an important meal and highlight their role in improving nutritional intake in children [9]. 

Studies on dietary quality, including selected food group intake, during the COVID-19 pandemic are limited to adult-focused studies. A report surveying 35 research organizations in Europe, North Africa, Western Asia, and the Americas showed that food consumption (types of food) and meal patterns (binge eating, snacking between meals, number of main meals) were more unhealthy during each country’s lockdown measures [29]. Australia showed a greater caloric intake due to increased snacking during lockdown measures [30]. The quality of meals consumed by Canadian students was reduced during the COVID-19 pandemic due to a decrease in the consumption of grains, fruits, vegetables, dairy, nuts, meat, and meat alternatives, while alcohol consumption significantly increased [31]. These findings support the results of our study. Conversely, healthier dietary behaviors were reported in a Spanish population due to greater adherence to traditional Mediterranean Diets during the COVID-19 lockdown [32]. 

In Japan, the proportion of children who had a “well-balanced dietary intake”, at least twice a day, was particularly low in lower-income households (Q4) compared to Q1, Q2, and Q3, before, during, and after the state of emergency. Furthermore, the proportion of those with a “well-balanced dietary intake” at least twice a day was notably low in both Q3 and Q4 during the state of emergency compared with before the declared state of emergency; the relative risk differences for each quartile were Q1: −19.0% (−19.6% to −18.4%), *p* < 0.001, Q2: −21.3% (−22.1% to −20.6%) *p* < 0.001, Q3: −25.4% (−26.1% to −24.7%), *p* < 0.001, and Q4: −34.8% (−35.6% to −34.0%), *p* < 0.001. The interaction *p* (vs. Q1) of Q2, Q3, and Q4 were all <0.001. According to a report from the Ministry of Health, Labour and Welfare of Japan, the poverty line in Japan for the year 2018 was 1.3 million JPY [20]. Therefore, Q4 falls into the household income level of near poverty. The ratio of people living below the poverty line was 15.4% for the year 2018 [20]. 

One possible reason for the disparities in dietary intake is that the school lunch has an important role in achieving adequate nutrient intakes for schoolchildren with low household incomes. Our previous studies of Japanese schoolchildren before the COVID−19 pandemic showed that children from low-income households had lower mean intakes of fish/shellfish, sugar, protein, and several micronutrients, higher intake of carbohydrates, and higher rates of nutrient shortages for vitamin B6, pantothenic acid, K, Mg, P, Fe, and Zn on days without a school lunch; however, the differences were not significant on days with a school lunch [16,17]. Therefore, due to the cessation of school lunches during the state of emergency [7], schoolchildren in low-income households had difficulties obtaining a “well-balanced dietary intake”, [21,22] which is designed to enhance the physical and mental growth of children [12,13]. Disparities in meal qualities among children in low-income households [14,15,16] continued to be observed before, during, and after the state of emergency, but especially during the state of emergency.

We also studied the relationship between household income and the guardians’ sense of burden for preparing meals. Analysis of the guardians’ subjective sense of burden after the COVID−19 state of emergency suggested that participants from low-income households had significantly less time to prepare meals, less psychological room to prepare meals, and a lesser financial position for choosing or consuming foods and meals when compared to the highest income households and before the state of emergency. One reason for this is that even after the state of emergency was declared over and school lunches were resumed [7], family meals, including lunches, were more likely to be prepared and consumed at home [33] due to continued restrictions and voluntary restraints on the use of facilities [34]. A previous online survey aimed at adults living in Italy during the pandemic reported that a high percentage of adults experienced: depressed mood, anxious feelings, hypochondria, and insomnia (61.3%, 70.4%, 46.2%, and 52.2%, respectively) during social isolation. Females were reportedly more anxious and disposed to comfort food than males; however, this study did not take economic disparities into account [35]. These studies suggest that guardians, especially females, are likely to be psychologically burdened by meal preparation during a pandemic.

There are plausible reasons why guardians from low-income households had significantly higher rates of having less time, psychological room, and financial position to prepare meals during the state of emergency. A previous study reported a low SES group, which was associated with a low school level, lacked knowledge of food, nutrition, health, and how they relate to each other when compared with higher SES groups [36]. Additionally, our results show that the prevalence of participants who answered “Economical circumstance of life after the state of emergency is worse than before the state of emergency” was highest in the low-income household group (Q1 (highest income level): 13.6%, Q2: 14.5%, Q3: 31.6%, and Q4 (lowest income level): 49.0%). The cost of food items affects the choice of purchase; previous studies have shown positive associations between the food cost per calorie and the energy densities of fruits, vegetables, meat, fish, and eggs, whereas the inverse associations have been reported for fat and sugar [37,38,39]. Thus, it is suggested that guardians in low-income groups tend to have a subjective sense of burden for preparing meals for schoolchildren after the COVID-19 state of emergency, such as having less time to prepare meals, less psychological room to prepare meals, and less financial position for choosing or consuming foods and meals. This sense of burden is suggested to contribute to inadequate dietary intake in children from low-income households and this could affect their physical and mental growth [12,13]. 

COVID-19 is still prevalent, and there are many issues to consider before the situation can be brought under control. Even though all household income levels showed poor quality of meals during the state of emergency, schoolchildren from low-income families had particularly insufficient dietary intakes compared with schoolchildren from high income families, and this remained the case throughout the pandemic. Meal quality after the state of emergency returned to the same levels as before the state of emergency; however, our study revealed that there was a higher prevalence of participants in the low-income household group who answered, “Economical circumstance of life after the state of emergency is worse than before the state of emergency. If the deterioration in living conditions among low-income families becomes long-term due to the continuation of the COVID-19 pandemic, schoolchildren in this group will have more difficulties in consuming the nutrients required for physical and mental growth, and guardians will feel more burdened with food preparation. To prevent the development of diseases and immune depression during pandemics, we must, on a continuous basis: avoid inadequate food supplies [40] and consume meals rich in essential micronutrients, including vitamins and minerals [41]. Establishing effective nutritional policies, such as continuous access to school lunches and food supplies even when schools are closed [42], are necessary to rectify schoolchildren’s socioeconomic disparities. Further studies are required to determine how the socioeconomic status of households affects the quality of meals for schoolchildren during the ongoing COVID-19 pandemic and how the intake of adequate food and nutrients can be achieved in schoolchildren, regardless of socioeconomic status.

The present study had several limitations: (i) we could not clarify the amount of food, energy, and nutrient intake in participants because this study was limited to searching for the prevalence of consuming selected food groups; (ii) inaccuracies due to self-reporting—previous studies suggest that obesity and the desire to lose weight may lead to under-reporting of energy intake, and a high BMI may lead to under-reporting of protein, sodium, and potassium intakes [43,44,45]; (iii) only 37% of participants completed the study, even though 51.7% of the children and their guardians initially agreed to participate. However, we used stratified two-stage clustering to select a nationally representative sample of the Japanese population and select for households with school children in the fifth (10–11 years old) and second grade of school (13–14 years old). We compared the percentage distribution of annual incomes in each participants’ household in 2020 with data from a representative set of Japanese households with children from the 2019 fiscal year [20]. The results were: 1.1 and 1.8% earned less than 1 million JPY; 3.2 and 4.4% earned 1 to less than 2 million JPY; 5.4 and 5.7% earned 2 to less than 3 million JPY; 7.7 and 6.4% earned 3 to less than 4 million JPY; 12.1 and 9.7% earned 4 to less than 5 million JPY; 11.4 and 12.4% earned 5 to less than 6 million JPY; 10.9 and 13.0% earned 6 to less than 7 million JPY; 12.1 and 11.0% earned 7 to less than 8 million JPY; 15.1 and 15.3% earned 8 to less than 10 million JPY; and 20.8 and 20.3% earned 10 or more million JPY, respectively. The percentage distribution of annual incomes in each participants’ household from this study was similar to data from the representative Japanese households in 2019.

In conclusion, our current study showed that household income was related to the quality of meals for schoolchildren before, during, and after the COVID-19 state of emergency was declared. We also found a relationship between household income and the guardians’ sense of burden for preparing meals after the state of emergency. Among Japanese schoolchildren, during the state of emergency, the prevalence of “well-balanced dietary intake”, defined as a meal containing both meat, fish, or eggs and vegetables, was lower than before the state of emergency but returned to the same prevalence in all household income levels. The number of schoolchildren receiving a “well-balanced dietary intake” was especially low in low-income households, before, during, and after the state of emergency, when compared with the highest-income households. In addition, the prevalence of “well-balanced dietary intake” notably dropped in the lowest household income group during the declared state of emergency when compared with before. Guardians from households with a low income reported significantly higher rates of having less time to prepare meals, less psychological room to prepare meals, and a lesser financial position for choosing or consuming foods and meals compared with before the state of emergency.

The evidence from our study suggests that a “well-balanced dietary intake” was not guaranteed in schoolchildren during the state of emergency and that school lunches are an important meal and system, contributing to the improvement of nutritional intake, especially in schoolchildren from low-income households. Changes are needed to enable all school children, regardless of socioeconomic status, to receive adequate food and nutrient intakes. Further studies are needed to examine the relationship between socioeconomic disparities and the quality of meals for schoolchildren during the ongoing COVID-19 pandemic.

## Figures and Tables

**Table 1:** Questionnaire to assess the prevalence of eating selected food groups in 10–14-year-old schoolchildren in Japan.

Please think back over the past month (December 2020). At home, did your child/children consume any of the following foods at least once a day? Please circle all foods that you eat at least once a day. (Circle all that apply) 1. Dairy products (milk, yogurt, cheese, etc.). 2. Meat, fish, and eggs. 3. Vegetables. 4. Fruits. 5. None of these; child does not eat these foods every day
Please think back over the emergency period (16 April to 13 May 2020). At home, did your child/children consume any of the following foods at least twice a day? Please circle all foods that you eat at least twice a day. (Circle all that apply) 1. Dairy products (milk, yogurt, cheese, etc.). 2. Meat, fish, and eggs. 3. Vegetables. 4. Fruits. 5. None of these; child does not eat these foods every day
Please think back to last year (December 2019). Please circle the foods that your child/children consume at least once a day at home. (Circle all that apply) 1. Dairy products (milk, yogurt, cheese, etc.). 2. Meat, fish, and eggs. 3. Vegetables. 4. Fruits. 5. None of these; child does not eat these foods every day
All schoolchildren are considered to be provided with 2. meat, fish, or eggs and 3. vegetables through their school lunches [8], except during a declared state of emergency.

**Table 2:** Characteristics of participants according to household income level: 10–14-year-old schoolchildren in Japan.

		Household Income Level								
		Q1 (Highest)		Q2		Q3		Q4 (Lowest)		
		N = 342		N = 251		N = 288		N = 230		*p* †
		N	%	N	%	N	%	N	%	
Gender	Boys	163	47.7%	137	54.6%	126	43.8%	119	51.9%	<0.001
	Girls	179	52.3%	114	45.4%	162	56.2%	111	48.1%	
Number of family members	2	22	6.4%	7	2.9%	15	5.4%	19	8.3%	<0.001
	3	74	21.6%	34	13.7%	37	12.8%	31	13.6%	
	4	184	53.8%	92	36.6%	132	45.9%	65	28.2%	
	5	46	13.6%	96	38.0%	62	21.4%	70	30.4%	
	6	9	2.5%	15	6.1%	34	11.8%	33	14.2%	
	7 and over	7	2.1%	7	2.8%	8	2.8%	12	5.4%	
Household annual income (million JPY)	<1	0	0.0%	0	0.0%	0	0.0%	12	5.2%	<0.001
	1 to <2	0	0.0%	0	0.0%	0	0.0%	36	15.5%	
	2 to <3	0	0.0%	0	0.0%	13	4.5%	46	20.3%	
	3 to <4	0	0.0%	4	1.7%	23	7.8%	59	25.8%	
	4 to <5	1	0.3%	9	3.3%	89	31.1%	36	15.7%	
	5 to <6	6	1.7%	42	16.7%	68	23.8%	11	4.8%	
	6 to <7	26	7.5%	57	22.8%	33	11.3%	6	2.7%	
	7 to <8	26	7.7%	69	27.5%	22	7.5%	18	7.7%	
	8 to <10	94	27.6%	35	13.9%	33	11.5%	6	2.5%	
	10 and over	189	55.2%	35	14.1%	7	2.5%	0	0.0%	
Economical circumstance of life after	Worse than before the state of emergency	46	13.6%	36	14.5%	91	31.6%	113	49.0%	<0.001
COVID-19 state of emergency	No different than before the state of emergency	279	81.5%	201	79.9%	188	65.3%	98	42.6%	
	Better than before the state of emergency	17	4.9%	9	3.7%	7	2.3%	6	2.6%	
	Do not want to answer	0	0.1%	5	1.9%	2	0.8%	13	5.8%	
Educational level of mother	Less than high school	2	0.5%	9	3.6%	8	2.9%	17	7.5%	<0.001
	High school	51	14.8%	52	20.8%	76	26.2%	87	37.9%	
	Vocational	58	17.0%	48	19.1%	50	17.2%	59	25.4%	
	Junior college	64	18.6%	57	22.7%	74	25.8%	41	17.8%	
	University/graduate school	166	48.5%	83	33.1%	78	27.2%	26	11.3%	
	No biological mother/Do not know/Do not want to answer	1	0.5%	2	0.8%	2	0.7%	0	0.1%	
Educational level of father	Less than high school	7	1.9%	10	4.0%	21	7.3%	25	10.8%	<0.001
	High school	67	19.5%	63	25.2%	99	34.4%	68	29.5%	
	Vocational	26	7.7%	30	12.0%	43	15.0%	29	12.6%	
	Junior college	2	0.6%	9	3.5%	5	1.8%	4	1.7%	
	University/graduate school	229	67.0%	135	53.8%	115	39.7%	68	29.8%	
	No biological father/Do not know/Do not want to answer	11	3.3%	4	1.4%	5	1.7%	36	15.7%	
Physique of child										
Height (cm)	Mean ± SD	150.7	±11.6	151.1	±11.0	149.8	10.8	152.6	±11.3	<0.001 ‡
Height percentile of Japanese	<3%	13	3.8%	8	3.3%	4	1.3%	13	5.5%	<0.001
children *	3–10%	21	6.2%	9	3.5%	22	7.8%	11	4.9%	
	10–25%	52	15.3%	52	20.6%	42	14.5%	31	13.7%	
	25–50%	75	22.0%	59	23.4%	83	28.6%	54	23.7%	
	50–75%	93	27.2%	58	23.2%	63	22.0%	62	26.7%	
	75–90%	48	14.1%	37	14.5%	47	16.4%	26	11.2%	
	90–97%	28	8.1%	13	5.2%	21	7.2%	23	9.8%	
	≥97%	12	3.3%	15	6.1%	6	2.2%	10	4.5%	
Weight (kg)	Mean ± SD	42.6	±10.8	42.0	±9.8	42.5	10.8	44.7	±11.9	<0.001 ‡
Weight percentile of Japanese	<3%	17	5.1%	5	2.1%	7	2.5%	6	2.4%	<0.001
children *	3–10%	32	9.3%	20	7.9%	29	9.9%	23	10.0%	
	10–25%	46	13.4%	53	21.1%	43	15.0%	36	15.7%	
	25–50%	88	25.9%	74	29.7%	70	24.2%	68	29.7%	
	50–75%	84	24.5%	64	25.3%	77	26.6%	44	19.1%	
	75–90%	54	15.9%	20	8.1%	32	11.2%	30	12.9%	
	90–97%	19	5.4%	11	4.4%	20	7.0%	14	6.0%	
	≥97%	2	0.5%	4	1.5%	10	3.5%	9	4.0%	
BMI (kg/m^2^)	Mean ± SD	18.5	±2.7	18.2	±2.6	18.7	3.0	19.1	±4.1	<0.001 ‡
BMI percentile of Japanese	<3%	23	6.6%	15	5.9%	10	3.5%	9	3.7%	<0.001
children *	3–10%	20	5.9%	14	5.6%	21	7.3%	19	8.2%	
	10–25%	55	16.1%	58	23.1%	54	18.6%	37	16.1%	
	25–50%	80	23.5%	63	25.0%	60	20.8%	68	29.6%	
	50–75%	86	25.2%	65	26.0%	74	25.6%	51	22.2%	
	75–90%	61	17.7%	22	8.9%	45	15.8%	22	9.8%	
	90–97%	17	4.9%	14	5.6%	18	6.2%	15	6.3%	
	≥97%	0	0.0%	0	0.1%	6	2.2%	9	4.1%	

† *p*-value for χ^2^ test among income levels. ‡ *p*-value for one-way analysis of variance test among income levels. * Height, weight, and BMI percentiles are the recommended values from The Japanese Society of Pediatric Endocrinology and The Japanese Association for Human Auxology [26,27,28]. Participants were weighted by the population of the municipality where each participant resides.

**Table 3:** Prevalence of intake of selected food groups at least twice a day according to household income level: 10–14-year-old schoolchildren in Japan before, during, and after COVID-19 state of emergency.

			Before the State of		During the State of		After the State of		
	Household		Emergency		Emergency		Emergency		
	Income Level		N	%	N	%	N	%	*p*
Milk and dairy products	Q1 (Highest)	(N = 342)	274	80.1%	171	49.9%	286	83.6%	<0.001
	Q2	(N = 251)	201	80.2%	141	56.1%	204	81.3%	<0.001
	Q3	(N = 288)	209	72.6%	126	43.9%	226	78.6%	<0.001
	Q4 (Lowest)	(N = 23)	156	67.9%	79	34.3%	162	70.5%	<0.001
Meat, fish, or eggs	Q1 (Highest)	(N = 342)	331	96.7%	285	83.5%	333	97.5%	<0.001
	Q2	(N = 251)	242	96.5%	210	83.8%	244	97.3%	<0.001
	Q3	(N = 288)	271	94.2%	230	79.7%	275	95.6%	<0.001
	Q4 (Lowest)	(N= 230)	216	94.1%	175	76.3%	222	96.3%	<0.001
Vegetables	Q1 (Highest)	(N = 342)	316	92.5%	276	80.7%	315	92.0%	<0.001
	Q2	(N = 251)	235	93.6%	207	82.5%	239	95.4%	<0.001
	Q3	(N = 288)	263	91.2%	208	72.2%	267	92.5%	<0.001
	Q4 (Lowest)	(N = 230)	207	90.0%	155	67.3%	205	89.3%	<0.001
Fruits	Q1 (Highest)	(N = 342)	131	38.3%	49	14.3%	145	42.3%	<0.001
	Q2	(N = 251)	81	32.3%	31	12.2%	100	39.7%	<0.001
	Q3	(N = 288)	78	27.1%	28	9.9%	100	34.8%	<0.001
	Q4 (Lowest)	(N = 230)	84	36.4%	30	13.1%	90	39.0%	<0.001
Do not eat any of the above	Q1 (Highest)	(N = 342)	3	0.8%	31	9.1%	1	0.4%	<0.001
	Q2	(N = 251)	4	1.4%	12	4.9%	2	0.8%	<0.001
	Q3	(N = 288)	11	3.9%	41	14.3%	9	3.2%	<0.001
	Q4 (Lowest)	(N = 230)	7	3.1%	33	14.3%	7	3.0%	<0.001

*p*-value for χ2 test among income levels. Participants were weighted by the population of the municipality where each participant resides.

**Table 4:** Poisson regression analysis of household income level and prevalence of “well-balanced dietary intake” at least twice a day: 10–14-year-old schoolchildren in Japan before, during, and after COVID-19 state of emergency.

Household		Before	During the State of			Interaction	After the State of			Interaction
Income		the State of	Emergency			*p*	Emergency			*p*
Level		Emergency	Relative risk Increase, % (95% CI)		*p*	(vs. Q1)	Relative Risk Increase, % (95% CI)		*p*	(vs. Q1)
Q1 (Highest)	Prevalence (%)	90.9%	75.2%				90.3%			
	Not adjusted	Ref	−19.0%	(−19.6% to −18.4%)	<0.001	-	−0.6%	(−1.2% to 0.0%)	0.042	-
	Adjusted †	Ref	−19.0%	(−19.6% to −18.4%)	<0.001	-	−0.6%	(−1.2% to 0.0%)	0.042	-
	Further adjusted ‡	Ref	−19.0%	(−19.6% to −18.4%)	<0.001	-	−0.6%	(−1.2% to 0.0%)	<0.001	-
Q2	Prevalence (%)	92.2%	74.4%				94.4%			
	Not adjusted	Ref	−21.3%	(−22.1% to −20.6%)	<0.001	<0.001	2.4%	(1.7% to 3.1%)	<0.001	<0.001
	Adjusted †	Ref	−21.3%	(−22.1% to −20.6%)	<0.001	<0.001	2.4%	(1.7% to 3.1%)	<0.001	<0.001
	Further adjusted ‡	Ref	−21.3%	(−22.1% to −20.6%)	<0.001	<0.001	2.4%	(1.7% to 3.1%)	<0.001	<0.001
Q3	Prevalence (%)	89.4%	69.3%				91.5%			
	Not adjusted	Ref	−25.4%	(−26.1% to −24.7%)	<0.001	<0.001	2.4%	(1.8% to 3.0%)	<0.001	<0.001
	Adjusted †	Ref	−25.4%	(−26.1% to −24.7%)	<0.001	<0.001	2.4%	(1.8% to 3.0%)	<0.001	<0.001
	Further adjusted ‡	Ref	−25.4%	(−26.1% to −24.7%)	<0.001	<0.001	2.4%	(1.8% to 3.0%)	<0.001	<0.001
Q4 (Lowest)	Prevalence (%)	87.9%	62.1%				89.1%			
	Not adjusted	Ref	−34.8%	(−35.6% to −34.0%)	<0.001	<0.001	1.3%	(0.6% to 2.0%)	<0.001	<0.001
	Adjusted †	Ref	−34.8%	(−35.6% to −34.0%)	<0.001	<0.001	1.3%	(0.6% to 2.0%)	<0.001	<0.001
	Further adjusted ‡	Ref	−34.8%	(−35.6% to −34.0%)	<0.001	<0.001	1.3%	(0.6% to 2.0%)	<0.001	<0.001

“Well-balanced dietary intake” was defined when both meat, fish, or eggs and vegetables were eaten. † Adjusted for sex (boys and girls), age (10–11 or 13–14 years old), and BMI (continuous). ‡ Adjusted for sex (boys and girls), age (10–11 or 13–14 years old, BMI (continuous), educational level of mother (less than high school, high school, vocational, college, and university/graduate school), and educational level of father (less than high school, high school, vocational, college, and university/graduate school). Participants were weighted by the population of the municipality where each participant resides.

**Table 5:** Poisson regression analysis of household income level and the guardian’s subjective sense of burden for preparing meals for 10–14-year-old schoolchildren in Japan after COVID-19 state of emergency.

	Household Income Level									
	Q1	Q2			Q3			Q4		
	(Highest)									
	N = 342	N = 251			N = 288			N = 230		
		Relative Risk Increase, % (95% CI)		*p*	Relative Risk Increase, % (95% CI)		*p*	Relative Risk Increase, % (95% CI)		*p*
**I have less time to prepare meals than before the state of emergency**										
Prevalence (%)	12.0%	10.8%			16.4%			15.6%		
Not adjusted	Ref	−10.9%	(−12.7% to −9.1%)	<0.001	31.3%	(29.8% to 32.9%)	<0.001	25.9%	(24.3% to 27.6%)	<0.001
Adjusted †	Ref	−11.7%	(−13.5% to −10.0%)	<0.001	31.4%	(29.9% to 33.0%)	<0.001	28.2%	(26.5% to 29.9%)	<0.001
Further adjusted ‡	Ref	−13.6%	(−15.4% to −11.8%)	<0.001	31.5%	(29.9% to 33.1%)	<0.001	18.4%	(16.6% to 20.1%)	<0.001
**I have more time to prepare meals than before the state of emergency**										
Prevalence (%)	23.8%	17.0%			16.0%			15.8%		
Not adjusted	Ref	−33.7%	(−35.0% to −32.3%)	<0.001	−40.1%	(−41.5% to −38.8%)	<0.001	−41.3%	(−42.8% to −39.9%)	<0.001
Adjusted †	Ref	−30.6%	(−32.0% to −29.3%)	<0.001	−44.0%	(−45.4% to −42.7%)	<0.001	−45.7%	(−47.1% to −44.2%)	<0.001
Further adjusted ‡	Ref	−22.1%	(−23.5% to −20.8%)	<0.001	−30.8%	(−32.2% to −29.4%)	<0.001	−22.8%	(−24.3% to −21.2%)	<0.001
**I have less psychological room to prepare meals**										
Prevalence (%)	12.6%	20.9%			19.5%			17.1%		
Not adjusted	Ref	50.5%	(49.1% to 52.0%)	<0.001	43.6%	(42.2% to 45.1%)	<0.001	30.5%	(28.9% to 32.1%)	<0.001
Adjusted †	Ref	50.2%	(48.7% to 51.7%)	<0.001	42.8%	(41.3% to 44.2%)	<0.001	33.1%	(31.5% to 34.7%)	<0.001
Further adjusted ‡	Ref	57.9%	(56.4% to 59.4%)	<0.001	56.5%	(55.0% to 58.0%)	<0.001	49.0%	(47.3% to 50.7%)	<0.001
**I have more psychological room to prepare meals**										
Prevalence (%)	17.1%	8.4%			9.6%			12.0%		
Not adjusted	Ref	−71.4%	(−73.2% to −69.6%)	<0.001	−58.4%	(−60.1% to −56.7%)	<0.001	−35.7%	(−37.3% to −34.0%)	<0.001
Adjusted †	Ref	−71.3%	(−73.1% to −69.4%)	<0.001	−59.0%	(−60.7% to −57.3%)	<0.001	−36.9%	(−38.6% to −35.3%)	<0.001
Further adjusted ‡	Ref	−58.9%	(−60.8% to −57.1%)	<0.001	−37.4%	(−39.1% to −35.7%)	<0.001	−11.9%	(−13.7% to −10.0%)	<0.001
**I have a lesser financial position for choosing or consuming foods and meals**										
Prevalence (%)	3.2%	8.3%			17.1%			32.9%		
Not adjusted	Ref	94.9%	(92.2% to 97.6%)	<0.001	167.4%	(165.0% to 169.8%)	<0.001	232.7%	(230.4% to 235.0%)	<0.001
Adjusted †	Ref	93.7%	(91.0% to 96.4%)	<0.001	167.5%	(165.1% to 169.9%)	<0.001	229.9%	(227.5% to 232.2%)	<0.001
Further adjusted ‡	Ref	78.7%	(76.0% to 81.4%)	<0.001	143.9%	(141.5% to 146.4%)	<0.001	194.0%	(191.6% to 196.4%)	<0.001
**Not Applicable to any above situation**										
Prevalence (%)	53.0%	54.8%			47.4%			44.5%		
Not adjusted	Ref	3.3%	(2.5% to 4.1%)	<0.001	−11.1%	(−11.9% to −10.3%)	<0.001	−17.5%	(−18.4% to −16.6%)	<0.001
Adjusted †	Ref	3.0%	(2.1% to 3.8%)	<0.001	−9.9%	(−10.7% to −9.1%)	<0.001	−18.1%	(−19.0% to −17.2%)	<0.001
Further adjusted ‡	Ref	0.6%	(−0.2% to 1.4%)	15.7%	−14.4%	(−15.3% to −13.6%)	<0.001	−22.4%	(−23.4% to −21.5%)	<0.001

† Adjusted for sex (boys and girls), age (10–11 or 13–14 years old), and BMI (continuous). ‡ Adjusted for sex (boys and girls), age (10–11 or 13–14 years old, BMI (continuous), educational level of mother (less than high school, high school, vocational, college, and university/graduate school), and educational level of father (less than high school, high school, vocational, college, and university/graduate school). Participants were weighted by the population of the municipality where each participant resides.

## Data Availability

The data are not publicly available due to privacy of participants.
